# Supplementary material for: Biodiversity Dynamics in a Ramsar Wetland: Assessing How Climate and Hydrology Shape the Distribution of Dominant Native and Alien Macrophytes
Source: Plants (Basel). 2025 Apr 3;14(7):1116. doi: 10.3390/plants14071116 (PMC11991433; doi:10.3390/plants14071116)
Supplement: Supplementary file 1 [file plants-14-01116-s001.zip › plants-3520681-supplementary.pdf]

plants

Article

Biodiversity Dynamics in a Ramsar Wetland: Assessing How Climate and Hydrology Shape the Distribution of Dominant Native and Alien Macrophytes

Fabio A. Labra [1,*] and Eduardo Jaramillo [2]

1 Centro de Investigación e Innovación en Cambio Climático, Facultad de Ciencias, Universidad Santo Tomás, Santiago 8370003, Chile
2 Instituto de Ciencias de la Tierra, Facultad de Ciencias, Universidad Austral de Chile, Valdivia 5090000, Chile; ejaramillo@uach.cl
* Correspondence: flabra@santotomas.cl

Supplementary Materials

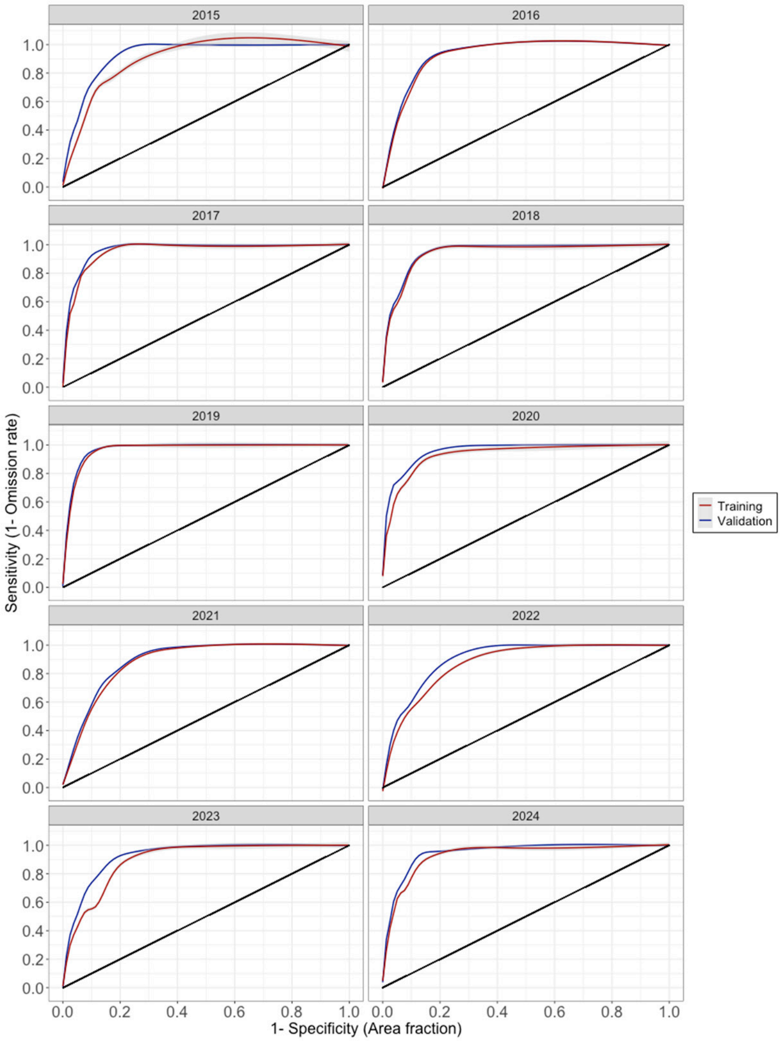

 https://doi.org/10.3390/plants14071116

Figure S1. Receiver-operator characteristic (ROC) curve for the distribution modeling of *Egeria densa* (in Spanish: Luchecillo) across the 2015-2024 ten-year period. The Figures illustrate the average ROC curve with a red line for the training subset of sites (averaged across the five cross-validations), and the average ROC curve with a blue line for the validation or test subset of sites. In both cases, the average value and 95% confidence interval illustrated are calculated using a general additive model (gam) in the R statistical computing environment (R Core Team 2023).

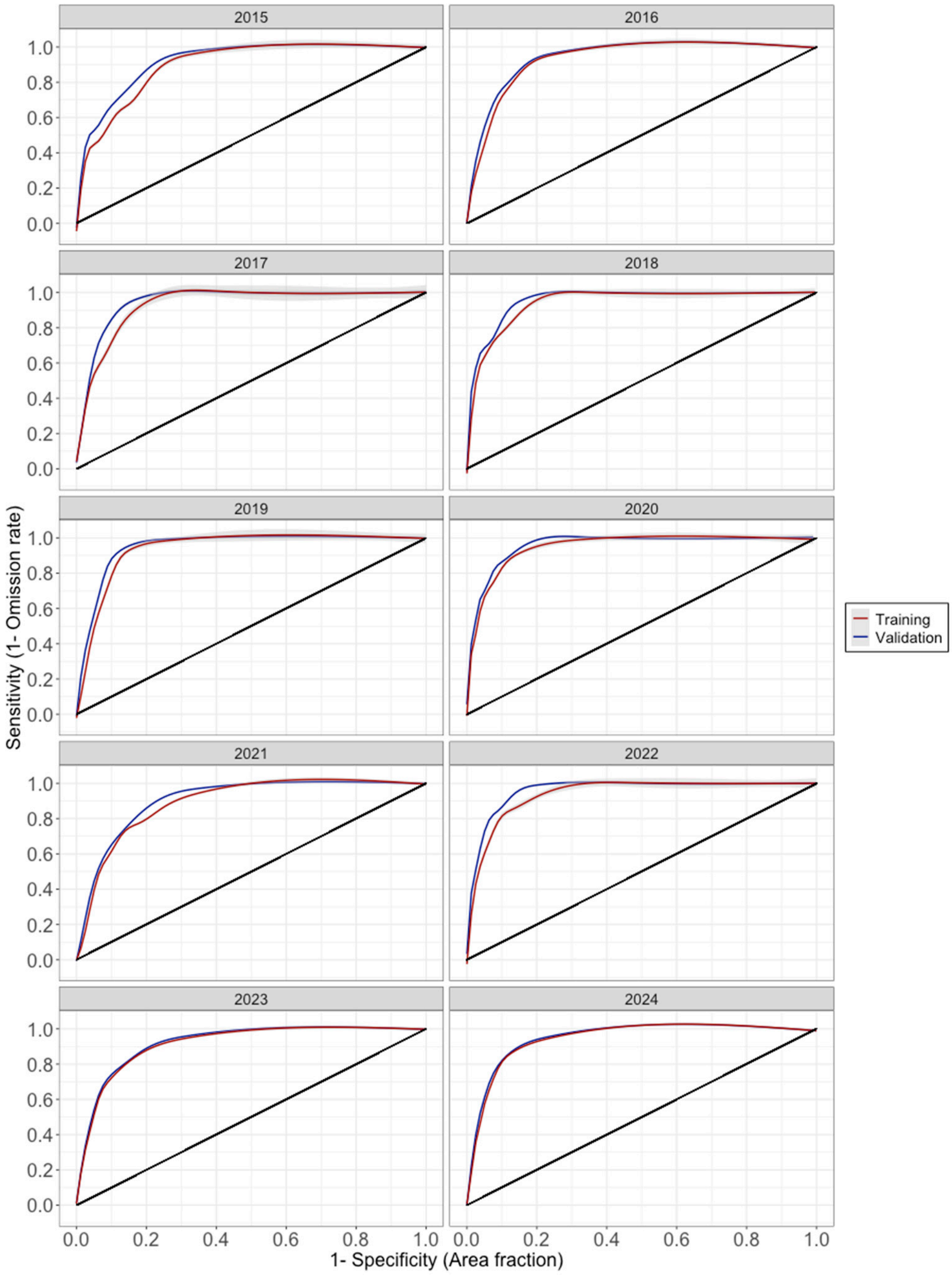

Figure S2. Receiver-operator characteristic (ROC) curve for the distribution modeling of *Schoenoplectus californicus* (in Spanish: Totora) across the 2015-2024 ten-year period. The Figures illustrate the average ROC curve with a red line for the training subset of sites (averaged across the five cross-validations), and the average ROC curve with a blue line for the validation or test subset of sites. In both cases, the average value and 95% confidence interval illustrated are calculated using a general additive model (gam) in the R statistical computing environment (R Core Team 2023).

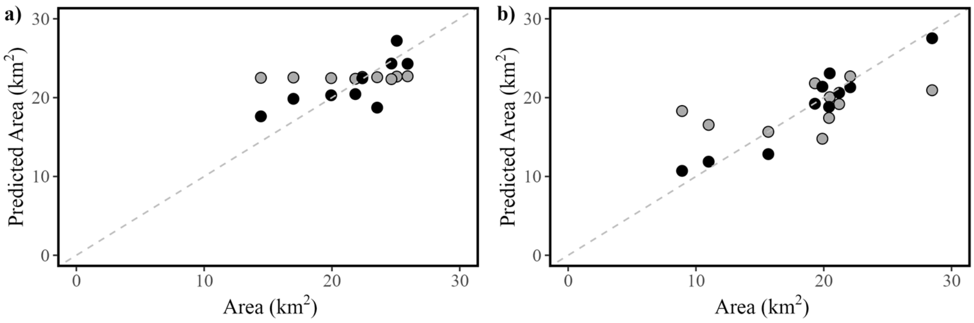

Figure S3. Observed *versus* Predicted area of suitable habitat for aquatic macrophytes in the RCW. The Figure shows the results for (**a**) *Egeria densa* and (**b**) *Schoenoplectus californicus* across the 2015-2024 ten-year period. The figures illustrate the model based on Year alone with grey filled circles, and the stepwise regression model prediction with filled black circles. The dashed line shows the line of slope 1, which corresponds to a perfect prediction.

(**a**)

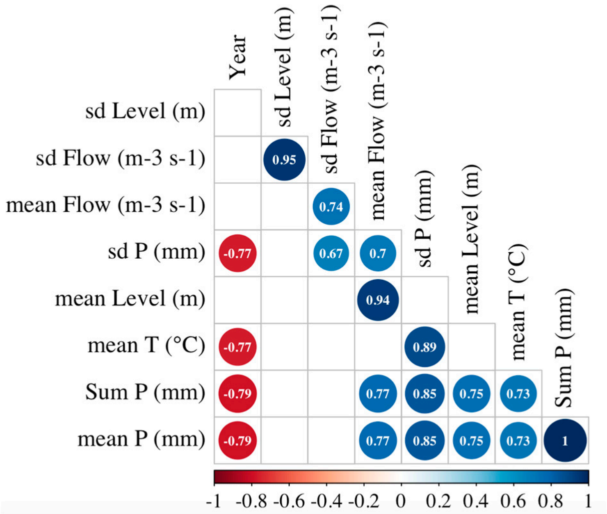

(**b**)

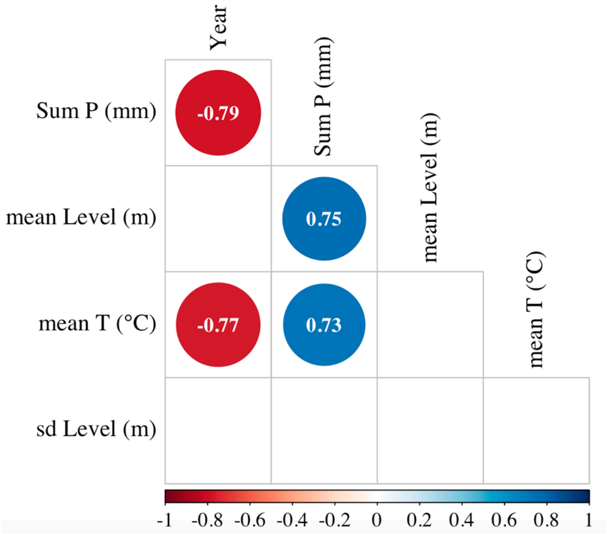

Figure S4. Correlation plot matrix for the climatic and hydrological drivers in the RCW. The figure shows (**a**) Correlation plot matrix for all climatic and hydrological drivers in the RCW. (**b**) Correlation plot matrix for the subset of climatic and hydrological drivers used to model the variability in areas of suitable habitats for *Egeria densa* and *Schoenoplectus californicus* across the 2015-2024 ten-year

period. The figures illustrate the significant Pearson correlation coefficient values, with circle size proportional to the correlation coefficient values as shown in the bottom color bar legend. Blank cells in the plot matrix, represent non-significant correlations.

Table S1. Comparison of the stepwise linear regression model and the reduced linear regression model evaluating the interannual variation in *Schoenoplectus californicus* suitable area within the RCW as a function of climatic and hydrological variables. The table shows results for a) Stepwise linear regression model for *Schoenoplectus californicus*, as shown in Table 3 and b) reduced linear regression model *Schoenoplectus californicus*. Selected predictor variables considered include Year, mean annual temperature (T_{Year} (°C), accumulated annual precipitation (sP_{Year} (mm)), mean annual water level (($Level_{Year}$ (m)), and annual water level standard deviation (s.d. Level (m)). The table shows Akaike Information Criterion values (AIC) for each model, as well as the fitted regression coefficients with their standard errors (β ± SE), t-Student values, and p-values for each predictor retained in the final model. Model fit statistics, including the adjusted R^2 and overall model significance, are also reported. The level of significance in each estimate is represented according to the following symbols: ns: p ≥ 0.05; *: p < 0.05; **: p < 0.01; ***: p < 0. 001.

Species and Variables	β ± SE	t	p-Value
a) *Schoenoplectus californicus:* Stepwise linear regression. AIC = 51.7			
Intercept	3072 ± 1395	2.20	0.093^{ns}
Year	-1.46 ± 0.68	-2.15	0.098^{ns}
T_{Year} (°C)	-6.19 ± 1.85	-3.35	0.029^{**}
sP_{Year} (mm)	0.02 ± 0.003	5.13	0.007^{***}
$Level_{Year}$ (m)	-46.47 ± 9.99	-4.65	0.010^{**}
s.d. Level (m)	14.95 ± 12.38	1.20	0.294^{ns}
b) *Schoenoplectus californicus:* reduced Stepwise linear regression. AIC = 55.4			
Intercept	78.52± 19.83	3.960	0.007^{***}
T_{Year} (°C)	-3.49± 1.45	-2.41	0.05^{*}
sP_{Year} (mm)	0.02 ± 0.003	4.76	0.003^{**}
$Level_{Year}$ (m)	-37.55± 10.28	-3.65	0.011^{*}

Table S2. List of herbarium specimens for *Egeria densa* and *Schoenoplectus californicus* in the RCW that have been deposited at CONC (Herbario de Concepción). The table includes the specimen number, scientific name, plant family, collection location, collection date, collector, and link to the herbarium database where the specimen is stored.

Specimen No.	Species	Family	Collection Location	Collection Date	Collector	Link
CONC 77814	*Egeria densa* Planch.	Hydrocharitaceae	Río Cruces, Fundo San Ramón, Valdivia	01/02/1967	Montero 7918	https://herbariodigital.cl/images/species/3408/
CONC 77809	*Egeria densa* Planch.	Hydrocharitaceae	Río Valdivia, Valdivia	01/11/1970	Montero 8463	https://herbariodigital.cl/images/species/3408/
CONC 108839	*Schoenoplectus californicus* (C.A. Mey.) Soják var. californicus	Cyperaceae	Valdivia	01/04/1958	Kunkel 10	https://www.herbariodigital.cl/catalog/details/6210/
CONC 109048	*Schoenoplectus californicus* (C.A. Mey.) Soják var. californicus	Cyperaceae	Valdivia	01/12/1961	Gunckel 37663	https://www.herbariodigital.cl/catalog/details/6210/

These specimens can be accessed through the Herbario Digital database, which provides digital images and metadata associated with these collections, including phenological information when available. The records for *Egeria densa* Planch. (Hydrocharitaceae) for the study area can be viewed at: https://herbariodigital.cl/images/species/3408/ (specimens CONC 77814 and CONC 77809). The records for *Schoenoplectus californicus* (Cyperaceae) for the study area can be viewed at: https://www.herbariodigital.cl/catalog/details/6210/ (specimens CONC 108839 and CONC 109048)

Disclaimer/Publisher's Note: The statements, opinions and data contained in all publications are solely those of the individual author(s) and contributor(s) and not of MDPI and/or the editor(s). MDPI and/or the editor(s) disclaim responsibility for any injury to people or property resulting from any ideas, methods, instructions or products referred to in the content.
